# An efficient electrode material for high performance solid-state hybrid supercapacitors based on a Cu/CuO/porous carbon nanofiber/TiO_2_ hybrid composite

**DOI:** 10.3762/bjnano.10.78

**Published:** 2019-04-01

**Authors:** Mamta Sham Lal, Thirugnanam Lavanya, Sundara Ramaprabhu

**Affiliations:** 1 Alternative Energy and Nanotechnology Laboratory (AENL), Nano Functional Materials Technology Centre (NFMTC), Department of Physics, Indian Institute of Technology Madras, Chennai-600036, India

**Keywords:** composite, electrochemical performance, porous carbon nanofiber, solid-state hybrid supercapacitor, supercapacitor, TiO_2_ nanoparticles

## Abstract

A Cu/CuO/porous carbon nanofiber/TiO_2_ (Cu/CuO/PCNF/TiO_2_) composite uniformly covered with TiO_2_ nanoparticles was synthesized by electrospinning and a simple hydrothermal technique. The synthesized composite exhibits a unique morphology and excellent supercapacitive performance, including both electric double layer and pseudo-capacitance behavior. Electrochemical measurements were performed by cyclic voltammetry, galvanostatic charge–discharge and electrochemical impedance spectroscopy. The highest specific capacitance value of 530 F g^−1^ at a current density of 1.5 A g^−1^ was obtained for the Cu/CuO/PCNF/TiO_2_ composite electrode in a three-electrode configuration. The solid-state hybrid supercapacitor (SSHSC) fabricated based on this composite exhibits a high specific capacitance value of 330 F g^−1^ at a current density of 1 A g^−1^ with 78.8% capacitance retention for up to 10,000 cycles. At the same time, a high energy density of 45.83 Wh kg^−1^ at a power density of 1.27 kW kg^−1^ was also realized. The developed electrode material provides new insight into ways to enhance the electrochemical properties of solid-state supercapacitors, based on the synergistic effect of porous carbon nanofibers, metal and metal oxide nanoparticles, which together open up new opportunities for energy storage and conversion applications.

## Introduction

To meet the rapidly growing demand for energy, more reliable, low cost, highly efficient and environmentally benign energy storage devices must be explored. Among many energy storage devices, supercapacitors are an ideal option for fast energy storage due to their high specific power (>10 kW kg^−1^), fast charge–discharge kinetics (in units of seconds), long cycle life (>10^5^), wide working potential and broad temperature range of operation [1–2]. The higher energy density and power density of supercapacitors are an important advantage over conventional dielectric capacitors and batteries. Supercapacitors can combine the advantages of batteries and conventional capacitors, which can store moderate energy as well as transport high power [3]. Thus, supercapacitors can potentially be used in many fields of application such as portable electronics, hybrid electric vehicles, pure electric vehicles, grid balancing, large-scale industrial equipment, and stand-by power systems [4–5]. Supercapacitors may be categorized by their energy storage mechanism into (i) electrochemical double-layer capacitors (EDLCs) and (ii) pseudo-supercapacitors. EDLCs, electrostatically store energy in a non-faradaic manner at the electrode–electrolyte interface, where only the physical processes are involved in separating the charges, and thus the surface area and porous nature of the electrode materials play the main role. However, charge stored only through electrostatic ion adsorption causes a relatively low energy density (<10 Wh kg^−1^), which leads to constrained performance. Thus, to achieve high performance EDLCs, we need to enhance the energy density without compromising power density. Pseudo-supercapacitors derive their capacitance from fast reversible faradaic reactions at the surface of electrode materials with the electrolyte, which stores a greater amount of charge than double layer supercapacitors and exhibits superior energy density [6]. Pseudo-supercapacitors have limited electrical conductivity and slow charge–discharge kinetics, resulting in a significant decrease in power density. Supercapacitors have been explored by realizing both faradaic and non-faradaic processes to store additional charges, moderate energy density, high power density and long service life [7–9]. Thus, it is important to combine pseudo-capacitance materials with a double-layer capacitor that can achieve the synergic advantages to meet all the requirements of upcoming energy storage devices.

Mainly porous, conductive, carbon-based materials, such as activated carbon, carbon black, carbon nanotubes, and graphene have been explored as electrode materials for EDLCs, which deliver high power density and prolonged cycle stability [10]. Among these, carbon nanofibers have been envisaged as a prospective electrode material due to its good mechanical strength, high surface area, relatively high conductivity [11–12]. Hence, carbon nanofibers produced by electrospinning, which is a cost-effective, simple and industry-viable technology, offer high production rate, high surface area and unique fiber pore structure [13]. These are potentially ideal electrode materials that can be widely used to fabricate high performance supercapacitors. The electrochemical performance of supercapacitors is defined by the type of electrolyte used. The electrolyte ion size should be proportional with the pore size of the active electrode materials [14]. The synthesis of carbon materials with very high surface area and appropriate pore size suitable for supercapacitor electrodes is a major challenge. It has been shown that this could be achieved by addition of sacrificial polymers, which decompose and offer the porous nature to the fiber during heat treatment [15]. CuO is one of the promising candidates for supercapacitors owning to its natural abundance, ecologically friendly and inexpensive characteristics. Moreover, Cu/CuO-based carbonaceous nanocomposites have emerged as an attractive and effective strategy that can enhance the electrical conductivity and provide relatively high specific capacity (increased by 10–30%). However, the electrochemical performance of this material deteriorates due to the large volume expansion during cycling. This results in a gradual loss of capacitance due to degradation of the electrode material, and thus poor electrochemical stability [16–18].

TiO_2_ nanoparticles loaded onto the carbon materials is a reasonable solution to overcome this challenge and is a potential electrode material for energy storage applications, ascribing to its low cost, long-term thermodynamic stability, abundance in nature, ecologically friendliness, photostability, and exceptional structural stability [19–21]. Thus, TiO_2_ nanoparticles can execute faradaic charge transfer reactions and provide the high cycle stability needed to enhance the supercapacitor behavior.

Herein, we report a novel approach for the fabrication of a Cu/CuO/porous carbon nanofiber (PCNF)/TiO_2_ (Cu/CuO/PCNF/TiO_2_) composite that is uniformly covered by TiO_2_ nanoparticles and is synthesized using the electrospinning method together with a hydrothermal technique, followed by air stabilization and carbonization processes to enhance the performance. More importantly, the obtained results have demonstrated that the composite material displays outstanding rate capability and long cycling stability which demonstrates its great potential as an efficient electrode material for supercapacitors. Overall, these composite materials offer a several advantages. First, the porous carbon of fiber morphology has a high aspect length-to-volume ratio and provides a reduced ion/electron diffusion path that allows fast, long-distance electron transport, which endows the necessary electrochemical performance. Second, Cu, which has good electrical conductivity and provides unique rate capabilities, was uniformly dispersed on the carbon matrices, which are extensively explored to meet the imperative requirements in the field of supercapacitors. Third, the introduction of nanoscale TiO_2_ can further enhance the energy density and specific power of supercapacitors owing to the pseudo-capacitance action of the metal oxide. Additionally, the uniform distribution of TiO_2_ nanoparticles on the surface of the Cu/CuO/PCNF composite material could provide strong mechanical strength to the composite that relegates capacitance fade and offers superior cycling stability. Our work introduces a new avenue and offers prospective electrode materials for advanced supercapacitors in the near future.

## Experimental

### Chemicals

Polyacrylonitrile (PAN, *M*_w_ = 150,000 g mol^−1^), polyvinylidene fluoride (PVDF, *M*_w_ = 120,000 g mol^−1^), titanium tetraisopropoxide (Ti{OCH(CH_3_)_2_}_4_, 97%), *N*,*N*-dimethylformamide (DMF, 99.8%), copper(II) nitrate trihydrate ((CuNO_3_)_2_·3H_2_O, 98%), 2-propanol (IPA, 99.5%) and *N*-methyl-2-pyrrolidinone (NMP, 99.5%) were purchased from Sigma-Aldrich and used as received without any further purification.

### Synthesis of Cu/porous carbon nanofibers (Cu/PCNF), PCNF and carbon nanofiber (CNF)

In a typical electrospinning process, initially 1 g of polyacrylonitrile (PAN) was dissolved in *N*,*N*-dimethylformamide (DMF) under magnetic stirring for 4 h at 50 °C to obtain a homogeneous transparent viscous solution. 20 wt % of copper(II) nitrate trihydrate [(CuNO_3_)_2_·3H_2_O] was added to this solution and stirred for further 30 min. The blended solution was loaded into a 10 mL disposable syringe with a needle diameter of 0.5 mm, pumped at a speed of 0.3 mL h^−1^ using a syringe pump. Aluminium foil was used as a collector, and the distance between tip of the needle and collector was maintained at 15 cm and a DC voltage of 17 kV was applied. The collected as-spun nanofiber mats were first air-stabilized at 280 °C for 2 h and carbonized at 800 °C in inert atmosphere to produce Cu/porous carbon nanofiber (Cu/PCNF). A similar procedure was followed to prepare the PCNF without Cu(NO_3_)_2_·3H_2_O and CNF were prepared without using (CuNO_3_)_2_·3H_2_O and PVDF.

### Synthesis of Cu/CuO/porous carbon nanofiber/TiO_2_ (Cu/CuO/PCNF/TiO_2_)

**Figure 1:**
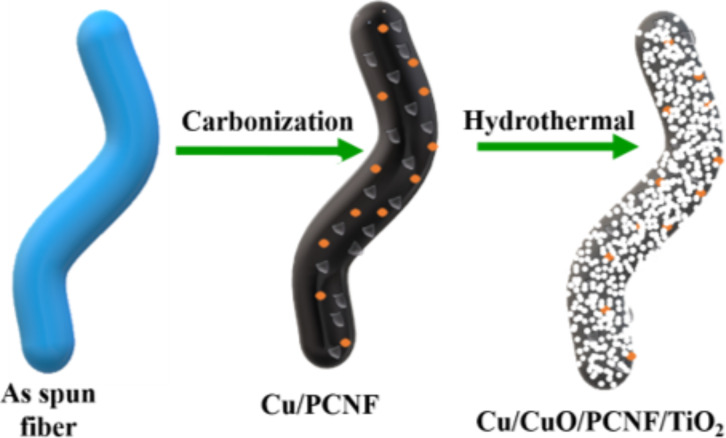
Schematic illustration shows the morphological changes during different synthesis steps.

A hydrothermal method was utilized to prepare Cu/CuO/PCNF/TiO_2_ composite material. Briefly, 0.1 g of prepared Cu/PCNF was dissolved in 50 mL of deionized water. Then, a dilute solution of titanium tetraisopropoxide (0.001 M) in isopropanol was added drop wise into the solution and the mixture was stirred for 30 min at room temperature. Afterwards, the desired amount of concentrated HNO_3_ was added to maintain a pH value of 3.5–4. Subsequently, it was heated at 180 °C for 12 h in a 100 mL teflon-lined autoclave. The treated powders were washed thoroughly with deionized water and ethanol for several times. The final product was filtered and dried overnight at 60 °C to get the Cu/CuO/PCNF/TiO_2_ composite. The formation of the composite from as-spun fiber during different steps of the synthesis is shown in Figure 1.

### Physical characterization

The surface morphology and elemental composition of the samples was investigated with an Inspect F50 field emission scanning electron microscope (FESEM) operating at 20 kV and a transmission electron microscope (JEOL JEM 2100) operating at 200 kV. The crystalline structure was identified using a Rigaku Rintz Ultima X-ray diffraction unit. Raman spectra were analyzed by a LabRAM HP 800 UV with a 632 nm He–Ne laser as the excitation source in the range of 100–3000 cm^−1^ at room temperature. The surface area and pore size analysis were performed using a Micromeritics ASAP 2020 instrument using Brunauer–Emmett–Teller (BET) adsorption theory. Degassing under high vacuum (10^−6^ mbar) at 200 °C was performed for samples before the surface area and pore size analysis. Thermogravimetric analysis was carried out using SDT Q600 with a TGA/DTA 6200 thermogravimetric analyzer from room temperature to 1000 °C under N_2_ flow.

### Electrochemical measurements

**Figure 2:**
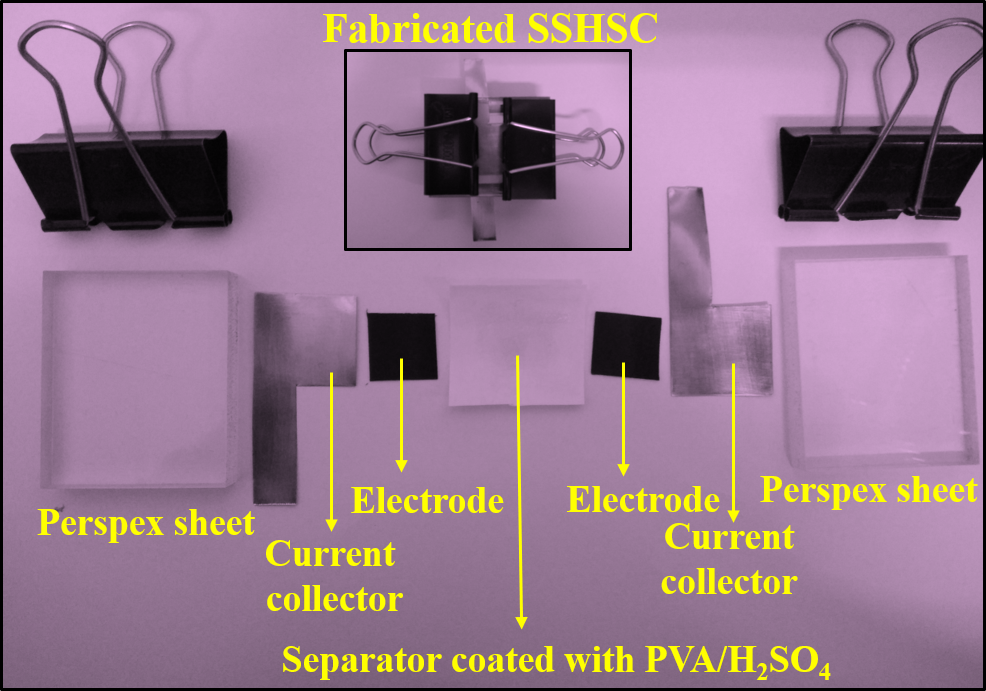
Photograph showing the different parts of fabricated symmetric solid-state hybrid supercapacitor (SSHSC).

A CH Instruments device (model CHI 900B) was employed to examine the electrochemical properties of the samples. The working electrode material slurry was prepared using a mixture of the material, i.e., polyvinylidene fluoride (PVDF) as binder (15%), conductive carbon (10%) and active material (75%) in *N*-methyl-2-pyrrolidone (NMP) solvent to form a viscous slurry using a mortar and pestle. This slurry was coated on the carbon sheet with an area of 1 cm^2^ and dried at 70 °C overnight and was used as the working electrode. A platinum wire acts as counter electrode and Ag/AgCl acts as reference electrode for the electrochemical measurements in three-electrode configuration in 1 M H_2_SO_4_ aqueous electrolyte from 0 to 1 V potential range. For the two-electrode configuration, a symmetric solid-state hybrid supercapacitor (SSHSC) was fabricated using the Cu/CuO/PCNF/TiO_2_ composite as an active electrode material coated on a carbon cloth (2 × 2 cm^2^), PVA/H_2_SO_4_ polymer gel electrolyte, separator of polypropylene sheet. The preparation of PVA/H_2_SO_4_ polymer gel electrolyte and the fabrication of SSHSC is given in Supporting Information File 1. Then, the assembly was placed between two Perspex sheets, along with stainless-steel plates as current collectors. An optical image showing different parts of the fabricated supercapacitors is given in Figure 2.

Cyclic voltammetry (CV) and galvanostatic charge–discharge (GCD) tests for the synthesized samples and its composites were performed over a voltage range from 0 to 0.8 V. The electrochemical impedance spectra (EIS) were recorded employing the same instrument over a frequency range of 100 kHz to 1 mHz with sine wave of amplitude 5 mV. The cycling stability test was performed at a current density of 5 A g^−1^ up to 10,000 charge–discharge cycles for the fabricated SSHSC.

The specific capacitance (*C*_m_) of the electrode for the Cu/CuO/PCNF/TiO_2_ composite can be calculated from the discharge profiles using the following equation [22–24]:

[1] $$C_{m}=\frac{I\times \Delta t}{m\times \Delta V}\;\;\text{for}\;\text{three-electrode}\;\text{configuration}$$

[2] $$C_{m}=2\frac{I\times \Delta t}{m\times \Delta V}\;\;\text{for}\;\text{two-electrode}\;\text{configuration}$$

where, *I* is the discharge current, ∆*t* is the discharge time, *m* is the mass of the active electrode material and Δ*V* is the potential window.

## Results and Discussion

**Figure 3:**
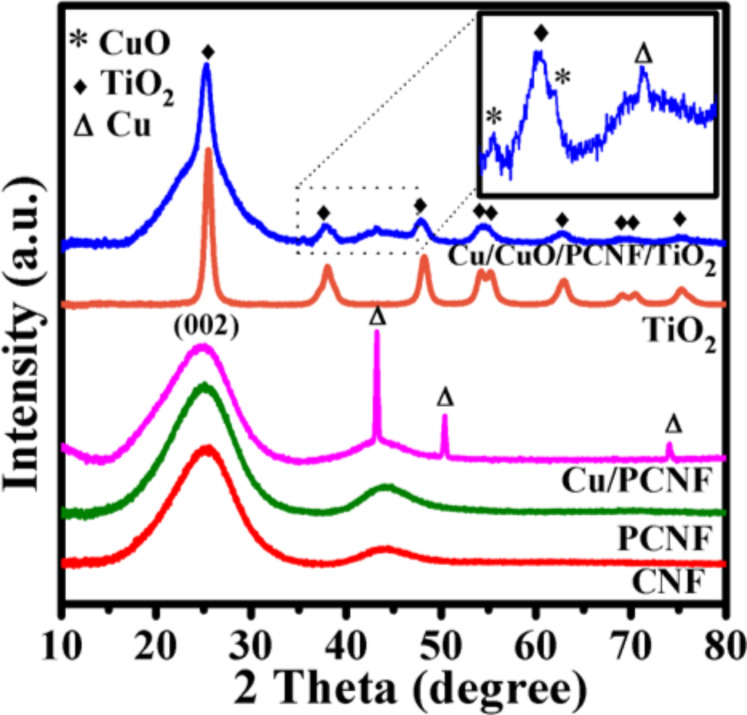
XRD spectra of CNF, PCNF, Cu/PCNF, TiO_2_ and Cu/CuO/PCNF/TiO_2_ composites.

The crystalline structure, purity and phase composition of the synthesized CNF, PCNF, Cu/PCNF, TiO_2_ and Cu/CuO/PCNF/TiO_2_ nanocomposites were examined by X-ray diffraction (XRD) as shown in Figure 3.

XRD pattern of CNF and PCNF shows the most relevant characteristic peaks at 2θ = 25.4°, 44.2° and 2θ = 25.1°, 44.0°, respectively, which correspond to (002) and (101) planes with d spacing of 0.350 nm and 0.354 nm, respectively. This clearly indicates the introduction of pores in PCNF during the decomposition of PVDF through the carbonization process. This process contributes to more disturbances and thus increases the d spacing from 0.350 nm to 0.354 nm. Also, the wide diffraction peak indicates the lower degree of graphitization. The diffraction pattern of Cu/PCNF could be indexed (002), (111), (200) and (220) respectively (JCPDS card # 00-003-1005). The sharp diffraction pattern of TiO_2_ assigned to the (101), (112), (200), (105), (211), (213), (116), (220) and (107) crystal planes of anatase TiO_2_ (JCPDS card # 00-001-0562) [25] and the similar crystal structure was observed in the Cu/CuO/PCNF/TiO_2_ composite material. Additionally, the Cu/CuO/PCNF/TiO_2_ composite shows (002), (101) planes of the carbon and (002), (111) planes of CuO. These results show that Cu nanoparticles on the surface were transform to CuO nanoparticles during hydrothermal synthesis, but in bulk of fiber, retained the phase of Cu.

**Figure 4:**
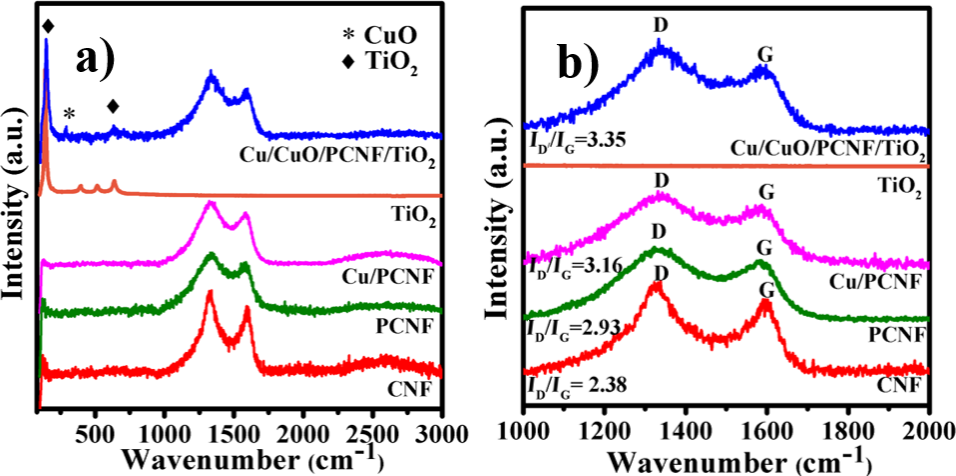
(a) Raman, spectra of CNF, PCNF, Cu/PCNF, TiO_2_ and Cu/CuO/PCNF/TiO_2_ composites and (b) Raman spectra of CNF, PCNF, Cu/PCNF, TiO_2_ and Cu/CuO/PCNF/TiO_2_ composites in the range of 1000–2000 cm^−1^.

Raman spectroscopy was used to estimate a crystalline phase and the degree of graphitization of the subsequent synthesized samples (Figure 4a).

CNF exhibited two characteristic peaks at 1587 and 1330 cm^−1^, corresponding to the G-band and D-band of typical carbonaceous materials, respectively. The D-band is related to disordered carbon, which shows vibrations with sp^3^ bonds in the crystal lattice defects which leads to disordered carbonaceous matrix. The G-band is assigned to the highly ordered graphite structure which arises due to stretching of carbon atoms bonded with sp^2^ bonds. TiO_2_ nanoparticles in the low frequency region were assigned to the E_1g_ (148.5 cm^−1^), B_1g_ (400 cm^−1^), A_1g_ (517 cm^−1^) and E_g_ (638 cm^−1^) modes of the anatase phase respectively [26]. A broad band at 297 cm^−1^ is observed in case of Cu/CuO/PCNF/TiO_2_ which is attributed to the A_g_ optical mode of CuO. Importantly, the composite Cu/CuO/PCNF/TiO_2_ showed the characteristic peaks of carbon, CuO and TiO_2_. Figure 4b shows the Raman spectra of CNF, PCNF, Cu/PCNF, TiO_2_ and Cu/CuO/PCNF/TiO_2_ composites in the range of 1000–2000 cm^−1^. The amorphous degree of the sample was calculated from the relative intensity ratio of the D- and G-bands (*I*_D_/*I*_G_) as 2.38, 2.93, 3.16 and 3.35 for CNF, PCNF, Cu/PCNF and Cu/CuO/PCNF/TiO_2_, respectively.

**Figure 5:**
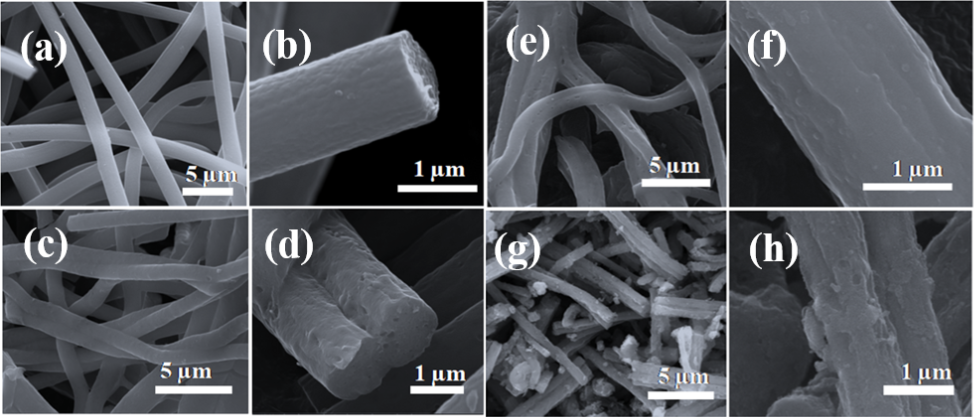
FE-SEM images of (a) CNF, (c) PCNF, (e) Cu/PCNF, (g) Cu/CuO/PCNF/TiO_2_ composites and (b), (d), (f) and (h) magnified images of CNF, PCNF, Cu/PCNF, and Cu/CuO/PCNF/TiO_2_ composites, respectively.

The morphology and microstructure of the as-prepared CNF, PCNF, Cu/PCNF and Cu/CuO/PCNF/TiO_2_ composite nanostructures were investigated by FE-SEM (Figure 5).

The FE-SEM images of the CNF nanofiber (Figure 5a and Figure 5b) carbonized at 800 °C shows smooth surfaces with no significant pores visible. Figure 5c shows the FE-SEM image of PCNF. Figure 5d shows the magnified image of PCNF which reveals the presence of pores in PCNF due to removal of PVDF during heat treatment. Figure 5e shows the FE-SEM image of Cu/PCNF and Figure 5f shows the magnified image of Cu/PCNF. Cu nanoparticles embedded due to the electrospinning process are visible on the surface of the Cu/PCNF material (Figure 5f). Figure 5g shows the FE-SEM image of Cu/CuO/PCNF/TiO_2_ composite and Figure 5h shows a magnified image. The uniform dispersion of TiO_2_ nanoparticles is clearly visible over the fiber surface after the hydrothermal treatment (Figure 5h). For energy dispersive X-ray spectroscopy elemental mapping of Cu/CuO/PCNF/TiO_2_, see Figure S1 in Supporting Information File 1.

**Figure 6:**
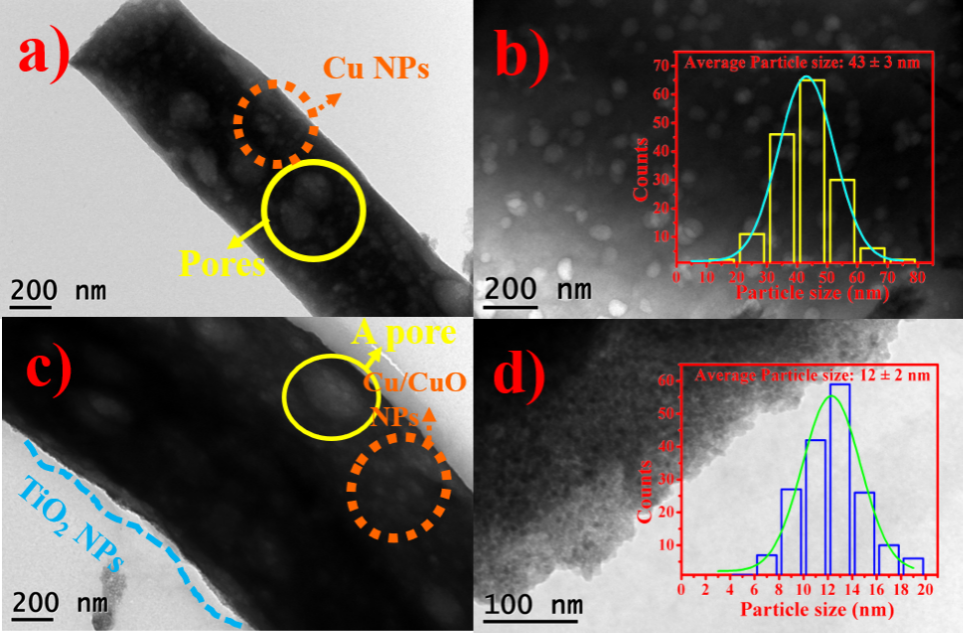
TEM image of (a) Cu/PCNF and (c) Cu/CuO/PCNF/TiO_2_ composites and (b, d) corresponding magnified images of (a, c). Inset (b, d) particle size distribution of Cu and TiO_2_, respectively.

Transmission electron microscope (TEM) observations of the Cu/PCNF and Cu/CuO/PCNF/TiO_2_ materials are shown in Figure 6.

For Cu/PCNF, the Cu particles were distributed within the carbon nanofiber networks, which is visible in Figure 6b. After the hydrothermal treatment, the TiO_2_ nanoparticles were uniformly covered on the surface of the Cu/CuO/PCNF/TiO_2_ composite, which is shown in Figure 6c. The average particle size of the Cu and TiO_2_ nanoparticles is about 43 and 12 nm (inset Figure 6b and Figure 6d).

**Figure 7:**
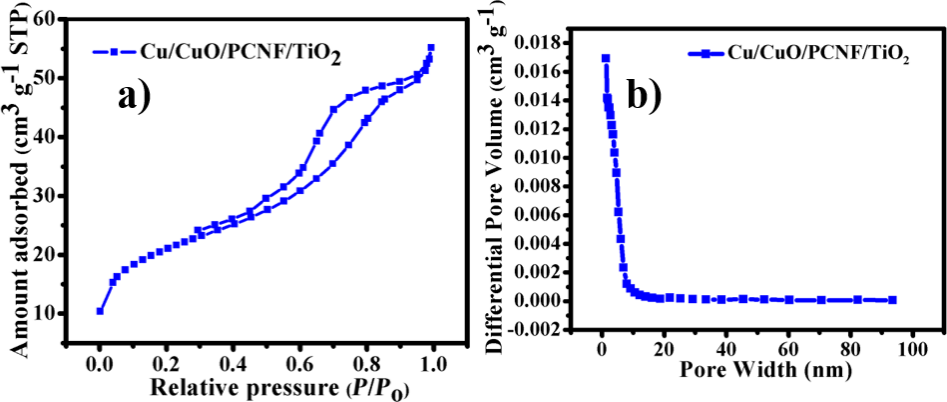
(a) N_2_ adsorption–desorption isotherms of the Cu/CuO/PCNF/TiO_2_ composite at 77 K and the (b) differential pore volume of the Cu/CuO/PCNF/TiO_2_ composite as a function of the pore diameter.

Furthermore, to study the porous structures and surface area, N_2_ adsorption–desorption isotherms and the pore size distribution were analyzed for the Cu/CuO/PCNF/TiO_2_ composites. To determine the performance of the supercapacitors, the surface area and pore size distribution are two important factors to provide good access of electrolyte to the electrode surface. Figure 7a shows the N_2_ adsorption–desorption isotherm with hysteresis loop from 0.3 to 1 *P*/*P*_0_ for the Cu/CuO/PCNF/TiO_2_ composite. The BET specific surface area of the Cu/CuO/PCNF/TiO_2_ composite was found to be 71.879 m^2^ g^−1^. Pores with a mean diameter of ≈12–15 nm were determined by the Barrett–Joyner–Halenda (BJH) method (Figure 7b).

**Figure 8:**
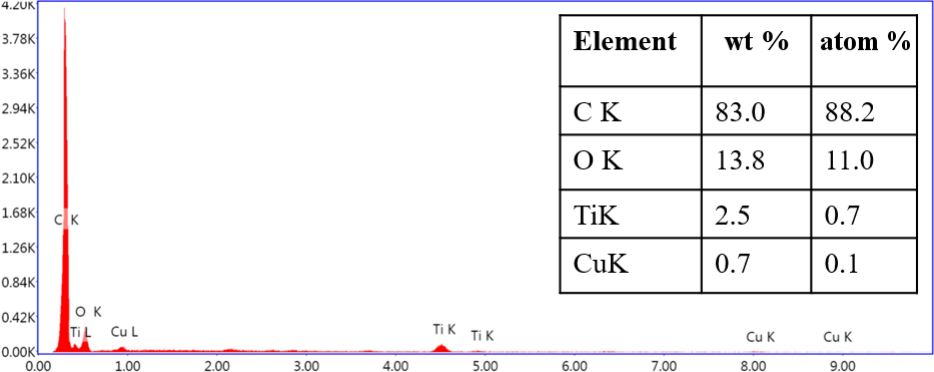
Energy dispersive X-ray spectrum of Cu/CuO/PCNF/TiO_2_.

The energy dispersive X-ray spectrum of the composite material shown in Figure 8 confirms the presence of C, O, Ti and Cu in the Cu/CuO/PCNF/TiO_2_ composite.

**Figure 9:**
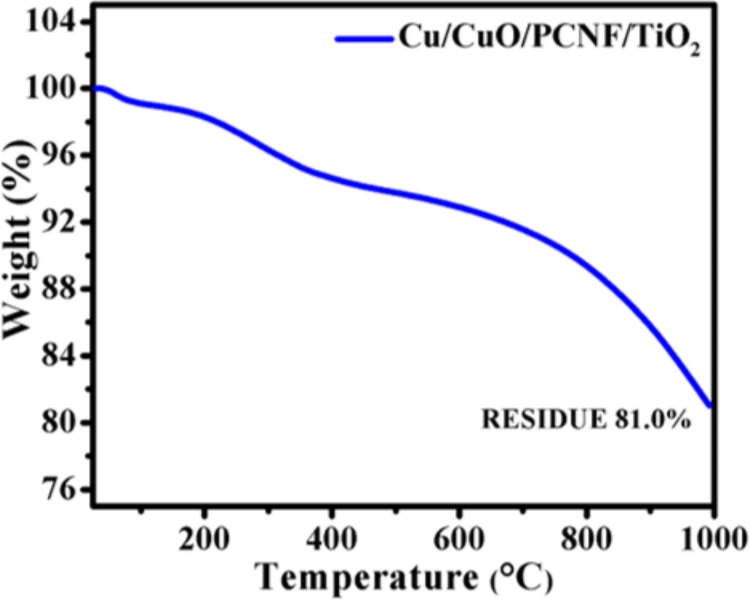
Thermogravimetric curve of the Cu/CuO/PCNF/TiO_2_ composite.

To study the thermal stability of the Cu/CuO/PCNF/TiO_2_ composite, TGA analysis was performed from room temperature to 1000 °C in inert atmosphere at a heating rate of 5 °C/min. Figure 9 shows the TGA curve of the Cu/CuO/PCNF/TiO_2_ composite.

The weight loss below 200 °C is due to the removal of the absorbed water molecules. The weight loss around 700 °C is due to the formation of rutile crystal structure. The composite material shows only 19% weight loss, which implies that the material has high thermal stability due to the uniform growth of the TiO_2_ nanoparticles on the fiber, providing high stability to the composite material.

### Electrochemical properties

The electrochemical studies of developed electrode materials for supercapacitors were executed by cyclic voltammetry, galvanostatic charge–discharge, and electrochemical impedance spectroscopy in a 1 M H_2_SO_4_ aqueous electrolyte in a three-electrode configuration.

**Figure 10:**
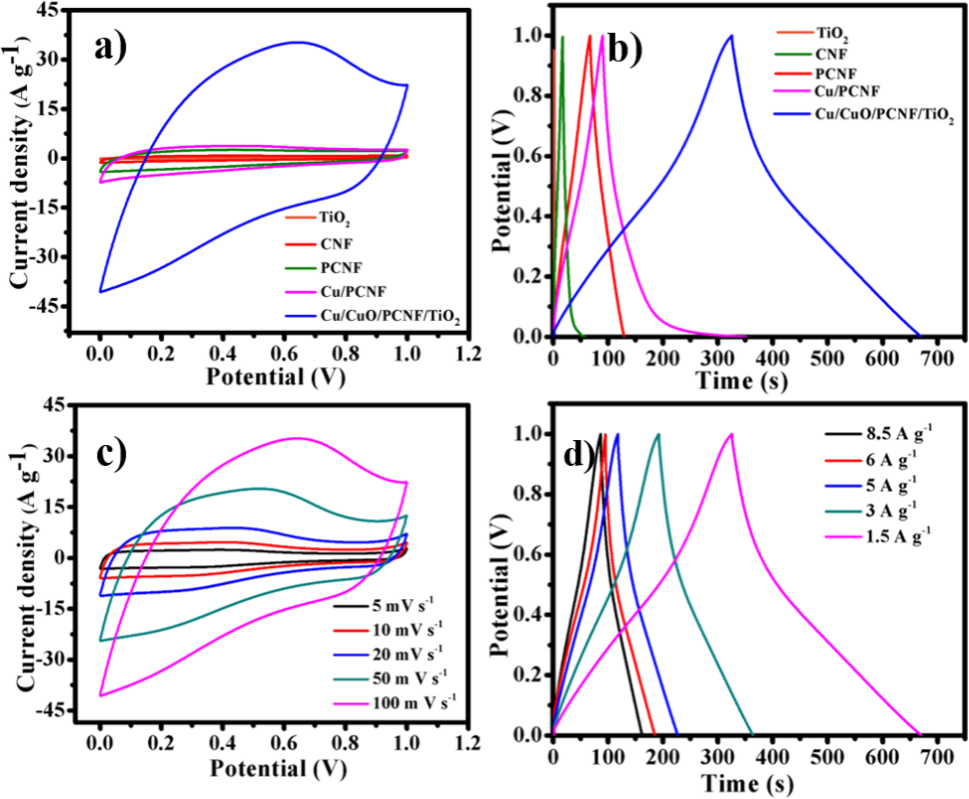
(a) Cyclic voltammetry (CV) curve, (b) galvanostatic charge–discharge (GCD) curve of TiO_2_, CNF, PCNF, Cu/PCNF and Cu/CuO/PCNF/TiO_2_ composites, respectively, (c) CV curves of Cu/CuO/PCNF/TiO_2_ composites at different scan rates and (d) GCD curves of Cu/CuO/PCNF/TiO_2_ composites at different current densities in a three-cell configuration.

Figure 10a shows the CV curves of TiO_2_, CNF, PCNF, Cu/PCNF and Cu/CuO/PCNF/TiO_2_ composite electrodes at a scan rate of 100 mV s^−1^ in the potential range (0–1 V). CNF, PCNF and Cu/PCNF samples show a quasi-rectangular box loop which represents electric double layer capacitance performance. Besides, the Cu/CuO/PCNF/TiO_2_ composite displays weak and broad characteristic peaks, which result from the redox reactions, indicating the pseudo-capacitive behavior of TiO_2_. Notably, the composite material shows a combined electric double-layer capacitance and pseudo-capacitive behavior with a higher integrated area compared to all the other electrode materials, assuring superior electrochemical performance of the Cu/CuO/PCNF/TiO_2_ composite electrode. To further elucidate the electrochemical behavior of the Cu/CuO/PCNF/TiO_2_ composite, different scan rates for CV tests were carried out which are shown in Figure 10c. To explore different potential ranges for the composite material Cu/CuO/PCNF/TiO_2_, CV tests were performed in potential range of −0.2 to 0.8 at different scan rates of 100, 50, 20, 10 and 5 mV s^−1^ (see Figure S2, Supporting Information File 1). No significant changes were observed upon changing the potential range from −0.2 to 0.8 V. To evaluate the specific capacitance of synthesized materials, galvanostatic charge–discharge measurements were performed (Figure 10b) at the current densities of 1.5 A g^−1^ in the potential range 0 to 1 V. All GCD curves of the electrode materials, except Cu/CuO/PCNF/TiO_2_, exhibit a symmetric triangular shape during the charging and discharging steps, indicating complete electric double layer behavior. The Cu/CuO/PCNF/TiO_2_ composite shows the long charge/discharge duration which results from the typical pseudo-capacitive performance of the TiO_2_ nanoparticles in the composite. The specific capacitance values of the TiO_2_, CNF, PCNF, Cu/PCNF and Cu/CuO/PCNF/TiO_2_ composite are calculated (according to Equation 1) as 1.26, 70, 97.5, 396 and 530 F g^−1^ respectively at a current density of 1.5 A g^−1^.

Nevertheless, the specific capacitance of the Cu/CuO/PCNF/TiO_2_ composite shows substantially higher values than all other electrode materials, which was due to the addition of TiO_2_ nanoparticles. TiO_2_ nanoparticles show pseudo-capacitive behavior due to faradaic charge transfer that takes place in redox reactions. Therefore, the supercapacitor performance of the Cu/CuO/PCNF/TiO_2_ composite electrode material results from both EDLC and pseudo-capacitance [27–28]. To explain the capacitive performance of the Cu/CuO/PCNF/TiO_2_ composite, additional GCD studies were performed at different current densities of 1.5, 3, 5, 6 and 8.5 A g^−1^ which is shown in Figure 10d. The effect of different TiO_2_ loadings on capacitance can be seen in Figure S3, Figure S4 and Figure S5 in Supporting Information File 1.

**Figure 11:**
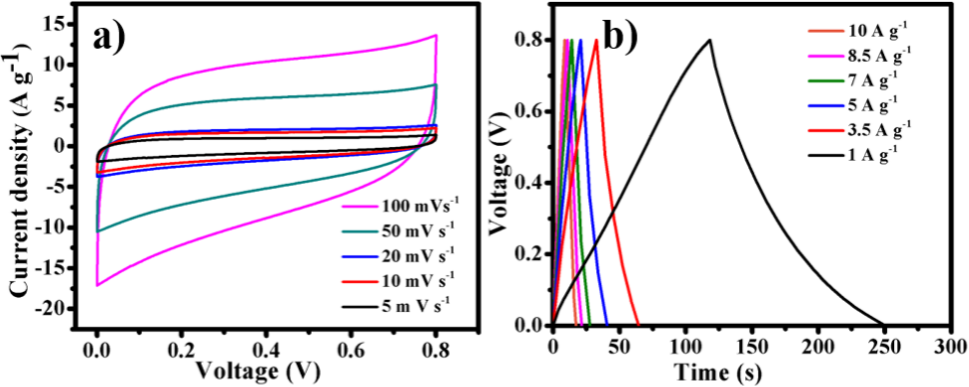
(a) CV curves for the fabricated SSHSC at different scan rates and (b**)** GCD curves of SSHSC at different current densities.

For a more realistic consideration, a practical cell construction was employed to fabricate the solid-state supercapacitor. A symmetrical two-electrode configuration was utilized to fabricate the solid-state hybrid supercapacitor (SSHSC) based on the Cu/CuO/PCNF/TiO_2_ composite, and electrochemical studies were performed in a PVA/H_2_SO_4_ polymer gel electrolyte. Figure 11a shows the CV profile of the SSHSC at different scan rates of 5, 10, 20, 50 and 100 mV s^−1^ in the voltage range from 0 to 0.8 V.

The fabricated SSHSC shows a highly rectangular area, which indicates improved capacitance performance for supercapacitor applications. Furthermore, the CV profiles of the SSHSC retain the rectangular shape, even at a high scan rate, which implies that good charge dissipation at the electrode surface offers a larger double layer interface, which provides greater capacitance to enhance the electrochemical performance. These results suggest that the fabricated SSHSC possesses higher specific capacitance, which confirms that the unique structure of the Cu/CuO/PCNF/TiO_2_ composite could provide a rapid charge-transfer capability to enhance the electrochemical performance. Noteworthy, we believe that the GCD method is a more efficient technique to find the capacitance value and the GCD profiles of SSHSC are shown in Figure 11b. The calculated GCD specific cell capacitance (according to Equation 2) for the SSHSCs are 330, 297.5, 262.5, 245, 233.7 and 200 F g^−1^ at a current density of 1, 3.5, 5, 7, 8.5 and 10 A g^−1^, respectively.

To the best of our knowledge, this is the best capacitance value ever achieved by solid state supercapacitors using metal oxide/carbon composite materials based on polymer gel electrolyte, and we compare the recently reported capacitance value, which is listed in Table 1 [29–37]. This may be attributed to the relatively high conductivity of Cu and CNFs, while nanoscale TiO_2_ helps to stabilize electrolyte transport and the mesoporous nature offers a high number of channels for faster ionic charge transport to enhance the capacitance performance of the Cu/CuO/PCNF/TiO_2_ composite material.

**Table 1:** Comparison of supercapacitive performance of fabricated SSHSCs with previous reports.

Solid state supercapacitor	Specific capacitance (F g^−1^)	Current density (A g^−1^)	Electrolyte	Energy density (Wh kg^−1^)	Ref.

NiMoO_4_–PANI	93	0.3	PVA/KOH	33.07	[29]
C/Na_2_SO_4_–PVA/C	38.6	5.0	Na_2_SO_4_/PVA	17.37	[30]
PAGH	170.6	1.0	PVA/H_2_SO_4_	26.5	[31]
CNTf–Mn-60	17	0.5	PYR_14_ TFSI	15.6	[32]
RCF_s_/MnO_2_/PEDOT	138	1.0	PVA/KCl	19.17	[33]
RuO_2_/CNO	305	1.0	PVA/H_2_SO_4_	10.59	[34]
graphene–WO_3_	171	1.0	PVA/H_2_SO_4_	26.7	[35]
MnO_2_@NiCo_2_O_4_/NC	110.6	1.0	PAAK/KOH	46.2	[36]
Co_3_O_4_–NF/CA	180.8	1.0	PVA/KOH	17.9	[37]
Cu/CuO/PCNF/TiO_2_	330	1	PVA/H_2_SO_4_	45.83	this work

**Figure 12:**
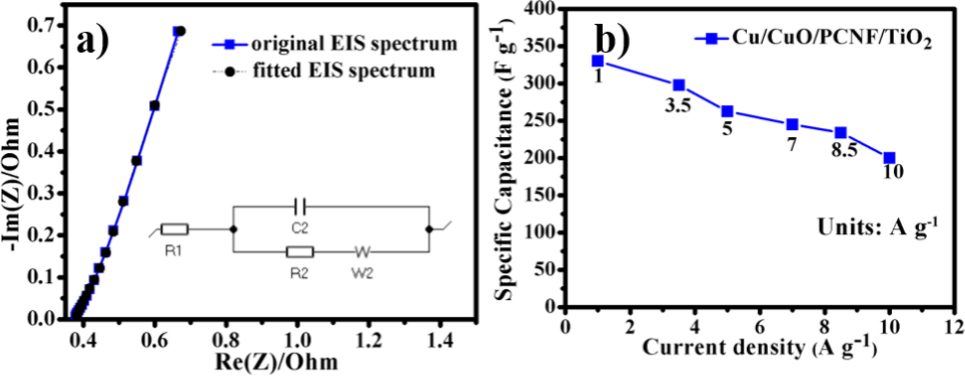
(a) Electrochemical impedance spectroscopy (EIS) spectrum of the SSHSC and (b) rate capability plot of the SSHSC at different current densities.

To study the internal resistance of the electrode and the electrode/electrolyte resistance of supercapacitors based on polymer gel electrolyte, electrochemical impedance spectroscopy (EIS) was employed in the frequency range of 100 kHz to 1 mHz at open circuit potential through applying a sine wave of amplitude 5 mV as shown in Figure 12a.

The SSHSC has smaller a semicircular arc diameter, suggesting the lower charge transfer resistance (R2) of the Cu/CuO/PCNF/TiO_2_ composite material. The calculated charge transfer resistance value is 15 Ω, which suggests that the presence of TiO_2_ nanoparticles on the surface of nanofibers could enhance the charge transfer performance. The ionic conductivity of the electrolyte system gleaned from R1 (solution resistances) values and the measured R1 value of the Cu/CuO/PCNF/TiO_2_ electrode is 0.37 Ω. The straight line in the low frequency region could be attributed to the good capacitive behavior of the SSHSC dominating the electrochemical double layer behavior. The rate capability of the SSHSC is emphasized in Figure 12b. We observed that the specific capacitance of the composite electrodes decreases with increase of the current density from 1 to 10 A g^−1^ yet retains the specific capacitance value of 200 F g^−1^ at 10 A g^−1^ with 61% of capacitance retention. This signifies the outstanding rate capability performance of SSHSC which makes it directly suitable for practical application.

**Figure 13:**
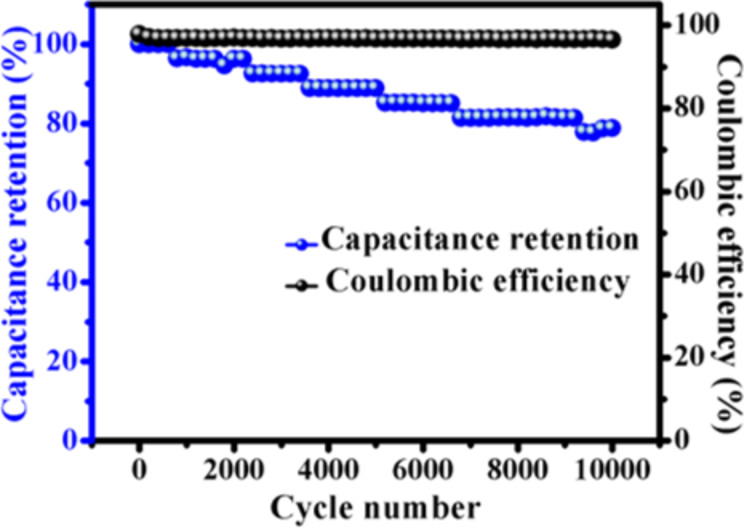
Capacitance retention and coulombic efficiency of the SSHSC at a current density of 5 A g^−1^.

Additionally, to predict the durability of the SSHSC, the electrochemical cycle stability was tested, which is crucial for practical application. Figure 13 shows the cycle stability performance of the SSHSC measured by GCD for 10,000 cycles at a current density of 5 A g^−1^. We observed a capacitance retention of 78.8% for the SSHSC over 10,000 cycles, which illustrates that the fabricated SSHSC based on a Cu/CuO/PCNF/TiO_2_ composite is appropriate for real energy applications. We have also calculated the coulombic efficiency and plotted it in the same figure (Figure 13). The coulombic efficiency remains at a stable high value of 96.7% up to 10,000 cycles.

The power density and energy density are two important parameters to completely determine the electrochemical performance of supercapacitors. We estimated the energy density (Wh kg^−1^) and power density (W kg^−1^) of the electrode material for a practical cell construction using the following equations

[3] $$E=\frac{1}{2}C_{m}{\left(\Delta V\right)}^{2}$$

[4] $$P=\frac{E}{\Delta t}$$

where *E* is the energy density (Wh kg^−1^), *C_m_* is the specific capacitance (F g^−1^), ∆*V* is the voltage window (V), *P* is the power density (W kg^−1^) and ∆*t* is the discharge time (s).

**Figure 14:**
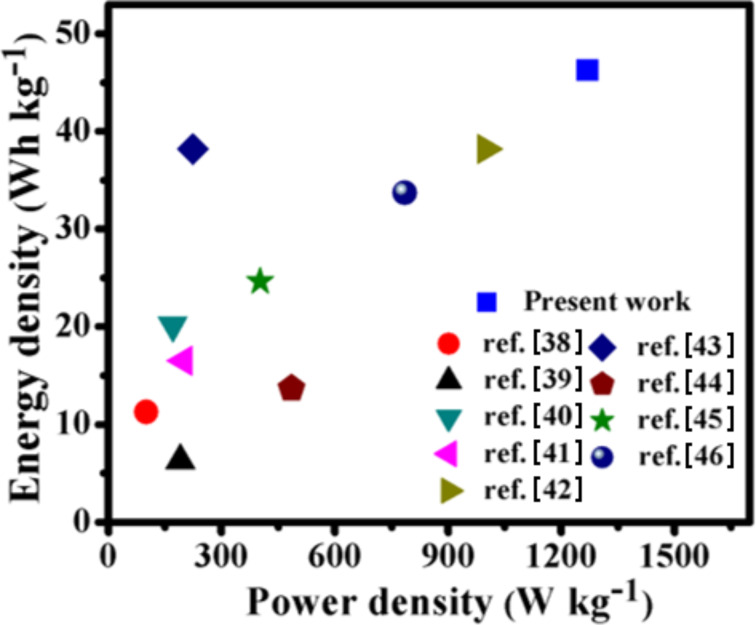
Ragone plot for the SSHSC and comparison with previous literature.

The Ragone plot clearly shows that a realistic SSHSC made by composite materials attains the highest energy density of 45.83 Wh kg^−1^ (calculated using Equation 3) at a power density of 1.27 kW kg^−1^ (calculated using Equation 4) at 0.8 V and 1 A g^−1^, respectively, which is superior to many of the earlier reported works (Figure 14) [38–46].

The superior supercapacitive behavior of the Cu/CuO/PCNF/TiO_2_ composite is attributed to the following aspects: (i) The fibrous morphology of CNF derived from PAN exhibits a high surface area and good conductivity which enhances the electrolytic ion diffusion and adsorption onto the electrode surface. (ii) A high number mesopores allows effective access to the electrolyte to the electrode surface due to easier ionic charge transport through the number of channels, thus improving the electrochemical properties of composite electrode material. (iii) The presence of Cu throughout the electrode effectively decreases the internal resistance and improves the conductivity and enhances the electrochemical reaction. The nanometer-sized CuO and TiO_2_ affords reduced ion diffusion length, which makes the composite attain easier electrolyte ion transfer and is expected to reduce the charge transfer resistance. Additionally, TiO_2_ has less volume expansion compared to other pseudo-capacitive materials, which provides the good cycling stability behavior to the SSHSC. Moreover, TiO_2_ nanoparticles with highly electrochemical active anatase phase could be ascribed to the extra capacitance enhancement which is evidenced by the CV and GCD measurements. Hence, we conclude that the suitable combination of electrode materials and its architecture could provide the large electrochemically available surface and proficient electron pathway, which is a promising approach for improving the electrochemical performance of SSHSCs.

## Conclusion

An efficient electrospinning technique and simple hydrothermal methods were used to prepare a Cu/CuO/PCNF/TiO_2_ composite material for supercapacitor applications. FE-SEM and TEM confirmed the uniform dispersion of TiO_2_ nanoparticles on the Cu/CuO/PCNF/TiO_2_ composite. The fabricated SSHSC manifested the specific capacitance of 330 F g^−1^ at a current density of 1 A g^−1^, which is mainly due to the combination of both pseudo-capacitance and double layer capacitance, which has a great effect on the enhanced electrochemical performance. Importantly, it is found that the fabricated SSHSC exhibits prolonged cycle stability with 78.8% retention even after 10,000 cycles. The fibrous morphology with good conductive and porous structure is beneficial for the fast charge transfer and electrolyte ion diffusion, which can assist the route to establish novel composites based solid state symmetric hybrid supercapacitors. Our synthesized composite based electrode can be expected to become a strong competitor for energy storage in hybrid electric vehicles.

## Supporting Information

File 1 Additional experimental data.
