# Supplementary material for: An efficient electrode material for high performance solid-state hybrid supercapacitors based on a Cu/CuO/porous carbon nanofiber/TiO2 hybrid composite
Source: Beilstein J Nanotechnol. 2019 Apr 1;10:781–93. doi: 10.3762/bjnano.10.78 (PMC6466681; doi:10.3762/bjnano.10.78)
Supplement: File 1 — Additional experimental data. [file Beilstein_J_Nanotechnol-10-781-s001.pdf]

BEILSTEIN JOURNAL OF NANOTECHNOLOGY

Supporting Information

for

An efficient electrode material for high performance solid-state hybrid supercapacitors based on a Cu/CuO/porous carbon nanofiber/TiO_2 hybrid composite

Mamta Sham Lal, Thirugnanam Lavanya and Sundara Ramaprabhu

Beilstein J. Nanotechnol. **2019,** *10,* 781–793. doi:10.3762/bjnano.10.78

Additional experimental data

License and Terms: This is a supporting information file under the terms of the Creative Commons Attribution License (http://creativecommons.org/licenses/by/4.0). Please note that the reuse, redistribution and reproduction in particular requires that the authors and source are credited.
The license is subject to the *Beilstein Journal of Nanotechnology* terms and conditions: (https://www.beilstein-journals.org/bjnano)

Preparation of PVA/H_2SO_4 gel electrolyte

To prepare PVA/H_2SO_4 electrolyte, in the first step 1 M H_2SO_4 (20 mL) aqueous solution is prepared. Then PVA (2 g) is added into 20 mL of 1 M H_2SO_4 solution to maintain 10 wt % of PVA. The obtained mixture is stirred continuously at 40 °C until a transparent gel is formed.

Fabrication of SSHSC

To fabricate SSHSC, first supercapacitor electrodes are coated with slurry with paint brush. The slurry is prepared in the manner as prepared for three-electrode configuration maintaining same weight percentage of PVDF binder, conductive carbon and active material. The slurry is coated on carbon cloth (2 cm × 2 cm) and vacuum dried at 70 °C for 12 h. The SSHSC fabrication requires two electrodes, a polypropylene sheet as separator, PVA/H_2SO_4 as gel electrolyte, stainless sheets as current collector, perpex sheet and binding clips. Coated carbon clothes are used as both positive and negative electrode. PVA/H_2SO_4 gel electrolyte is coated on both sides of separator with brush and pressed between two electrodes. Further, this assembly is sandwiched between current collectors along with perpex sheets. Finally, two big size clips are used to bind the fabricated SSHSC for mechanical stability.

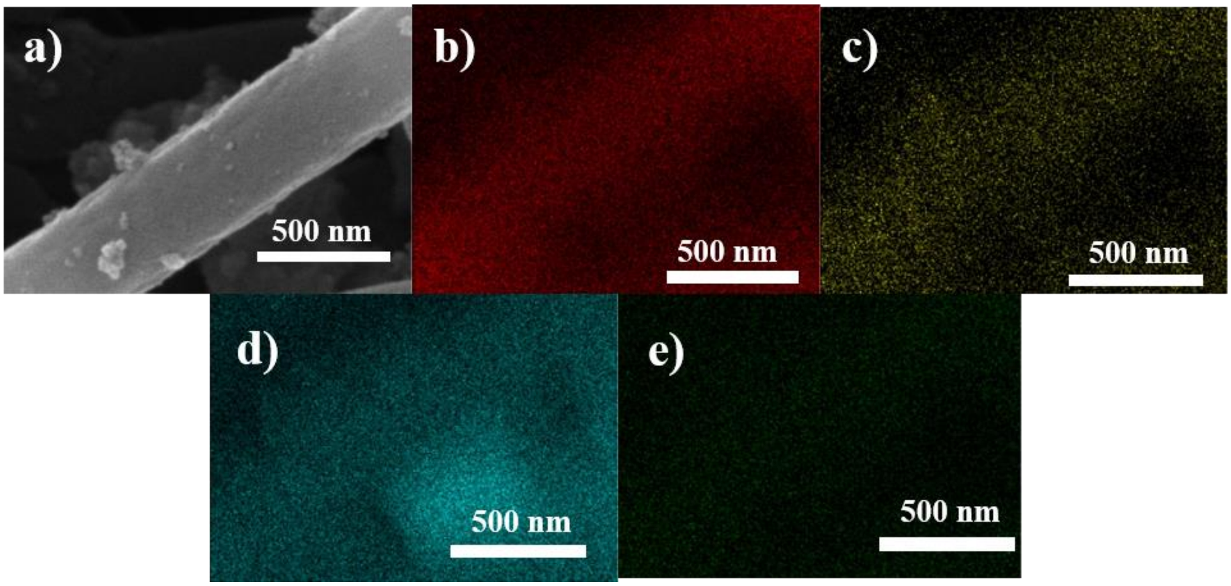

Figure S1: Typical EDX elemental mapping graphs of a) $Cu/CuO/PCNF/TiO_2$, b) C K, c) O K, d) TiK and e) CuK. The scale bar is 500 nm

Half-cell studies of Cu/CuO/PCNF/TiO_2 composite

CV test for Cu/CuO/PCNF/TiO_2 composite in the potential range −0.2 to 0.8 for three-electrode configuration were performed using 1 M H_2SO_4 aqueous electrolyte at different scan rates of 5, 10, 20, 50 and 100 mV s^{-1} (Figure S2).

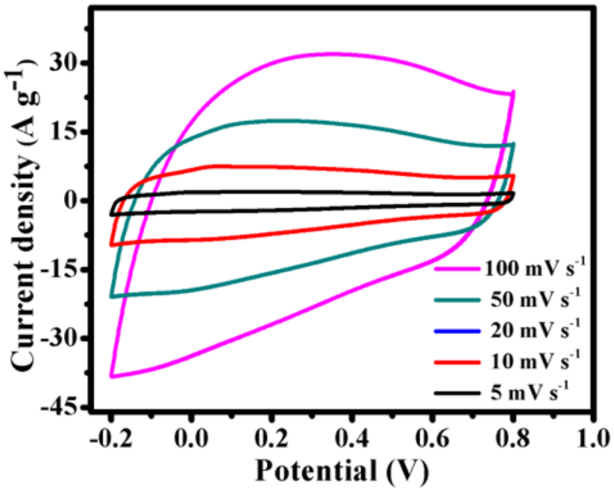

Figure S2: CV curves of Cu/CuO/PCNF/TiO_2 composite in three-electrode configuration at different scan rates in 1 M H_2SO_4.

Effect of amount of TiO_2 on capacitance

To study the effect of amount of TiO_2 on capacitance, we synthesized three different composite materials by varying concentration of titanium tetraisopropoxide 0.001, 0.003 and 0.005 M and labelled as C0001, C0003 and C0005, respectively. In result, as amount of TiO_2 increase, the capacitance values decrease.

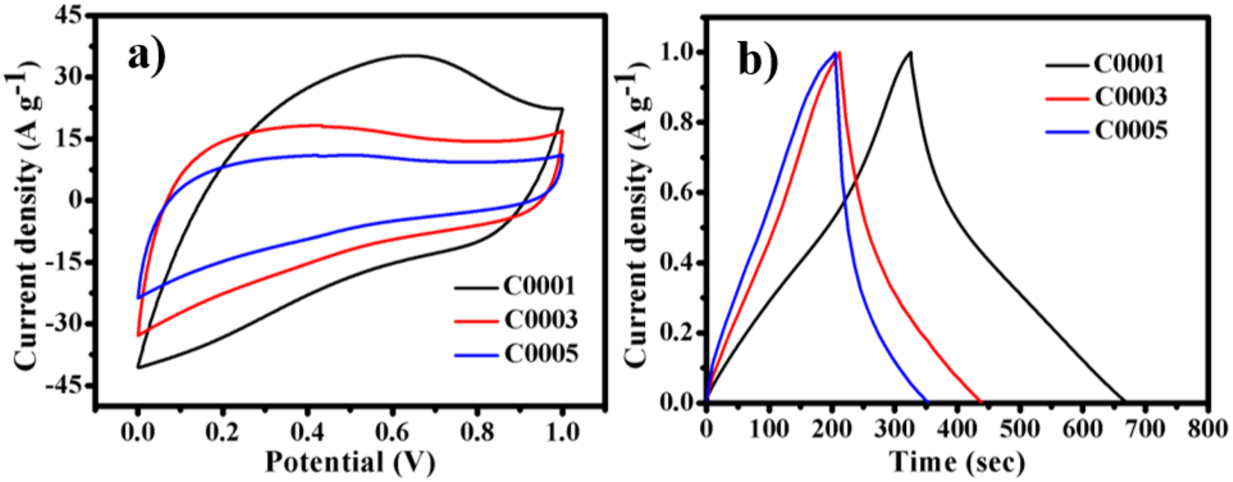

Figure S3: (a) CV and (b) GCD curves of C0001, C0003 and C0005, respectively.

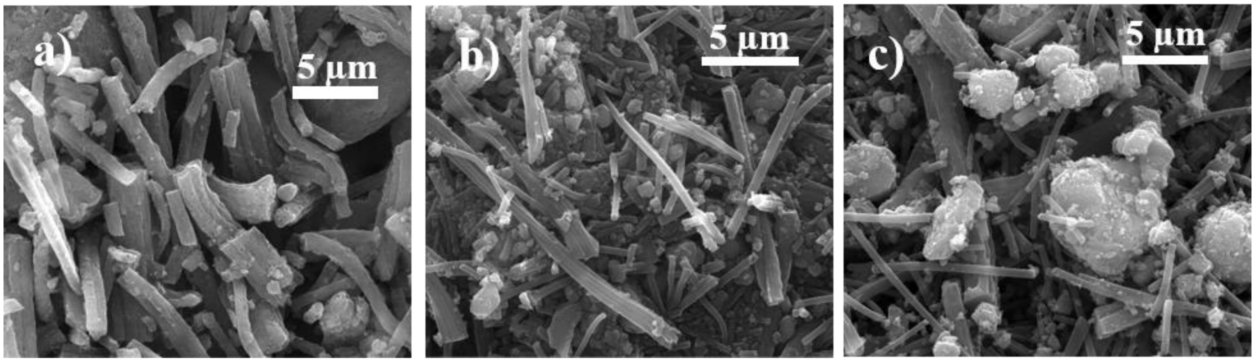

Figure S4: FE-SEM images of a) C0001, b) C0003 and c) C0005.

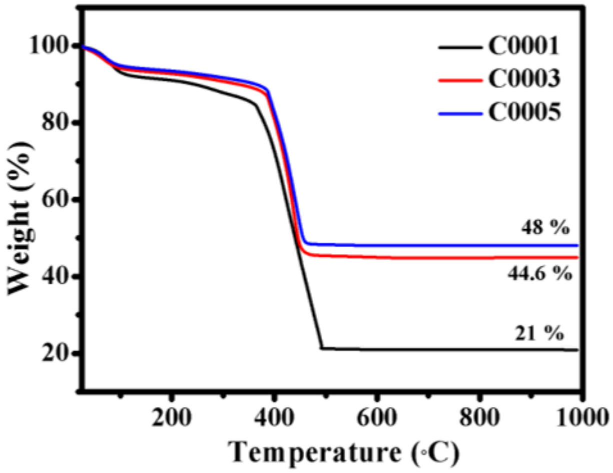

Figure S5: TGA curves for C0001, C0003 and C0005.
